# Regenerative medicine therapies: lessons from the kidney

**DOI:** 10.1016/j.cophys.2019.12.008

**Published:** 2020-04

**Authors:** Jamie A Davies, Patricia Murray, Bettina Wilm

**Affiliations:** 1 Deanery of Biomedical Sciences, University of Edinburgh, EH8 9XB, Edinburgh, UK; 2 Department of Cellular and Molecular Physiology, University of Liverpool, UK; 3 Centre for Preclinical Imaging, University of Liverpool, UK

## Abstract

We focus on three strategies for renal regenerative medicine; administering cells to replace damaged tissue, promoting endogenous regeneration, and growing stem cell-derived organs. Mouse kidney regeneration can be promoted by stem cells injected into the circulation which do not become new kidney tissue but seem to secrete regeneration-promoting humoral factors. This argues against direct replacement but encourages developing pharmacological stimulators of endogenous regeneration. Simple ‘kidneys’ have been made from stem cells, but there is a large gap between what has been achieved and a useful transplantable organ. Most current work aims to stimulate endogenous regeneration or to grow new organs but much remains to be done; misplaced hype about short-term prospects of regenerative medicine helps neither researchers nor patients.

**Current Opinion in Physiology** 2020, **14**:41–47

This review comes from a themed issue on **Regeneration**

Edited by **Catherina G Becker**, **Thomas Becker** and **Joseph C Wu**

For a complete overview see the Issue and the Editorial

Available online 30th December 2019

**https://doi.org/10.1016/j.cophys.2019.12.008**

2468-8673/© 2020 The Authors. Published by Elsevier Ltd. This is an open access article under the CC BY license (http://creativecommons.org/licenses/by/4.0/).

## Introduction and scope

In a medical context, ‘regeneration’ encompasses at least two concepts; recreation of a healthy tissue from a damaged tissue *in vivo*, and *in vitro* creation of a new tissue or organ from a patient’s stem or progenitor cells to replace a damaged or missing version. The first of these, regeneration of healthy tissue from injured tissue, takes place naturally in many organs: a rodent liver surgically reduced to one third of its original size will, for example, regenerate the missing mass within a week [1]. Indeed, generation of new healthy tissue from an undamaged side of the liver can be triggered surgically by deliberate induction of clotting in an already injured zone. Several hypotheses have been suggested to explain this effect, including availability of oxygen and nutrients from the increased blood flowing to the healthy area when the alternative route is closed, and responses in healthy tissue to injury-stimulated increase in IL6, HGF, TGFα, TNFα and other cytokines (the competing theories are reviewed by Ref. [2]). However it works, the effect is now used clinically to promote growth before resection of the damaged zone [3].

Unfortunately, some organs do not regenerate well naturally, at least not well enough to restore health in common human diseases. Kidneys, for example, can recover from some insults but serious renal disease tends to become chronic and function is lost over time. Axons of the mammalian central nervous system show very little natural regenerative capacity [4], resulting in permanent and serious disabilities from spinal injuries and strokes. Comparison of tissues that regenerate after injury with those that do not, together with experimental manipulation of pathways, suggests that non-regeneration may be not so much failure of surviving cells to react at all to injury, but rather a reaction in a direction other than regeneration, the usual other direction being toward fibrosis [5]. Inflammation, in particular, has been identified as a potent influence on the balance between regeneration, local fibrosis, and spreading fibrosis and further injury [6].

**Figure 1:**
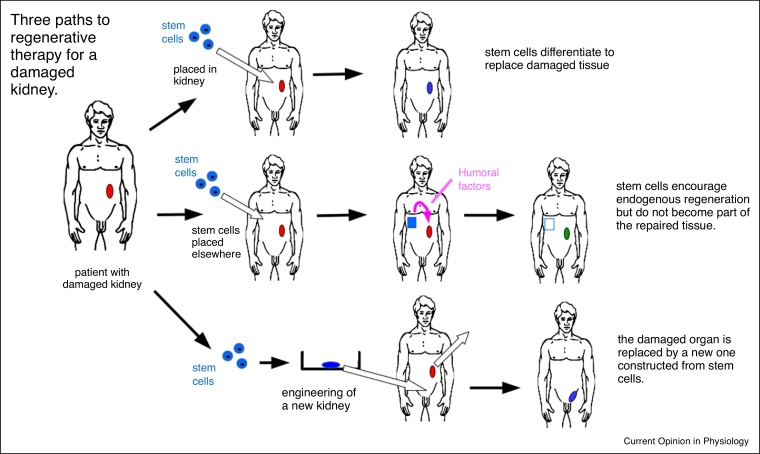
The three types of regenerative medicine therapy discussed in this article, illustrated with reference to the kidney. The top row depicts direct application of stem cells to the damaged organ, in which they differentiate to make cells to replace the damaged tissue. The middle row depicts grafting of stem cells to another site in the body (perhaps in a container permeable to molecules not cells), where they secrete humoral factors that drive the cells of the natural kidney to regenerate the tissue. The bottom row depicts construction of a replacement kidney *in vitro*, followed by transplantation; this might be transplantation of a full-sized kidney, as depicted, or of an engineered rudiment that will grow *in situ*. In all rows, the stem cells might be patient-derived, with or without correction of underlying genetic defects.

Increasing understanding of the regeneration-inflammation-fibrosis axis, together with improved techniques for manipulating stem cells, is fueling intense research into ways to promote regeneration. In this review, we will focus on progress in three areas (Figure 1); administration of cells to contribute directly to a regenerating tissue, the possibility of identifying and developing pharmacological mimics of regeneration-promoting humoral factors, and construction of new tissues for transplantation. Given limitations of space and the scale of the literature, we will use one organ, the kidney, as an exemplar. Most of the principles can be applied to other organs and tissues.

## Anatomy and organization of the kidney

Human kidneys are excretory organs approximately 10 cm high and 5 cm broad and deep, located in the upper lumbar region of the back, one each side of, and a little ventral to, the spinal column. Internally, their structure is dominated by epithelial tubes. Some of these tubes are blood vessels; blood enters the middle of the organ via the renal artery and then follows a tree-like system of arteries that deliver it to about one million fine arterioles in the outer part (cortex) of the kidney. Each arteriole feeds a knotted, porous capillary bed, the glomerulus, which allows water and small molecules to pass from the capillary, through a filter, to the inside of an epithelial tube, the nephron. Each nephron tube consists of a series of specialized segments; the proximal convoluted tubule is a long tube folded into a small volume in the cortex, and its cells are very active in both recovering solutes such as salts, sugars and amino acids from the filtrate, and in actively secreting organic acids and bases from the extracellular fluid into the filtrate. The transport systems involved can concentrate drug or toxin metabolites in these cells, a common cause of kidney damage. The next segment of the nephron is the loop of Henle, which dips down, hairpin-like, into the inner part of the kidney (medulla). Salt recovery from the loop makes this medulla very salty. The nephron then returns to the cortex where it leads, via the distal convoluted tubule, to a branch of the tree-like urine collecting duct system. The filtrate passes along this collecting duct system, down through the salty medulla again, where the saltiness is used to drive water recovery along an osmotic gradient, and eventually down into a urine-collecting basin, the renal pelvis, in the centre of the kidney. From there, it leaves the organ via the ureter. The blood components that did not pass through the glomerular filter passes, via another loop into the medulla and back to collect recovered water, into the renal vein and thence out of the organ.

From even the very brief outline above, it will be clear that in the kidney, function depends critically on anatomical arrangement. The blood system must interact precisely with the nephron system, the segments of the nephron system have to follow in a correct sequence, and all systems need to have their components located properly in the cortex-medulla axis for osmotic gradients to work properly. If a damaged kidney is to be regenerated, the new tissue will have to have this very high degree of organization; just having metabolically active cells present in a random arrangement will not do.

## The goal of using stem cells to replace damaged tissue *in situ*

Research in stem or progenitor cells has naturally led to the idea of applying cells to patients for the purposes of regenerating missing or damaged tissue. The common assumption underlying this aim is that stem cells will produce differentiated progeny that will replace missing tissue. This approach is based on the natural role of stem or progenitor cells as a pool tapped into by the organ for homeostasis and endogenous repair. In the kidney, however, defining renal stem or progenitor cells remains a challenge [7,8^•^,^9^,^10^]. Furthermore, isolating stem or progenitor cells for therapies has not been possible since kidney stem cells that can integrate and regenerate all types of kidney cells have been elusive so far. Mouse models of lineage tracing have suggested that progenitor cells of proximal tubules of the nephrons exist [11,12], and they have been reported to reside in the papilla or near the glomeruli [13,14]. In principle, it might therefore be possible to use single renal progenitor cell types to regenerate specific parts of the kidney, or to use a mixture for multiple tissues.

Another approach is to abandon ‘adult’ progenitor cell types, and use cells with the pluripotency associated with the early embryo. Protocols have been published to drive embryonic stem cells (ESCs) and induced pluripotent stem cells (iPSCs) to form entire renal structures *in vitro*, and some of these structures show evidence of limited renal function when transplanted to an *in vivo* location [15,50,16]. However, there is no convincing evidence from animal models that renal stem or progenitor cells have successfully integrated and replaced damaged renal tissue. Furthermore, human cell-based therapies have not been approved for clinical use to treat kidney disease (https://hsci.harvard.edu/faq/kidney).

## The goal of using stimulating endogenous regeneration via paracrine/humoral factors or their mimics

A surprising by-product of work intended to build new tissue *in situ* directly from stem cells has been the discover that repair and regeneration after cell administration in the kidneys and other organs can be accomplished by paracrine means [17^•^]. In particular the use of mesenchymal stromal cells (MSCs) as cell therapies in rodent models has shown that the cells have repair or regenerative capacity even when they never reach the kidneys [18,19]. Recent studies have revealed that the underlying mechanism may be death of the administered cells, which results in stimulation of the immune system, which in turn protects from or resolves the kidney damage [17^•^,^20^]. The molecular and cellular details of this process are still obscure, although it is becoming clear the macrophages and T-cells, as well as neutrophils, play a role [17^•^,^19^]. Furthermore, MSC-derived vesicles, including extracellular vesicles, might be involved in the communication between the administered cell therapies, the damaged organ tissue and immune system [21,22].

The observation that MSCs can promote regeneration indirectly and over large distances raises the hope that the critical mediators of MSC-immune-regeneration interactions may be soluble molecules or molecules that can be prepared in vesicles. If these natural molecules could be identified, then it may be possible to mimic their actions pharmacologically either by classical small-molecule drugs or more complex biologics. There is already a large database of drugs that modulate the immune system [23], and it may be possible that, when the target molecules for promotion of immune-mediated renal regeneration are identified, it will be found that relevant drugs already exist.

## Safety and efficacy of regenerative medicine therapies

The injection or administration of stem or progenitor cells to treat damaged kidneys is associated with a range of questions which have implications for the efficacy, but importantly also the safety, of potential therapies: Which is the best administration route? What is the fate of the administered cells after administration? Will the cells home to the injured kidney, or also lodge at non-target tissue sites where they could form tumours?

These questions raise important issues of cell-based regenerative medicine therapies: not only do the administered cells have to convey efficacy in repair or regeneration, they also need to be safe, and not pose risks of uncontrolled growth. We have recently undertaken a comprehensive preclinical study to address the question of safety of cell therapies, using a unique set of preclinical imaging modalities to track the cells *in vivo* [24]. Our results have shown that different cell types, including human bone marrow-derived and umbilical cord-derived MSCs, never reach the kidneys when administered via the intravenous route since the cells are sequestered in the lungs. Injection into the left ventricle of the heart allowed the administered cells to distribute widely in the body, including accumulation in the kidneys. However, both routes of administration led to death of the cells within 48 hours within the lungs or kidneys. An important observation of our study was the fact that after intravenous administration of umbilical cord-derived MSCs, which are already used in human trials, we could observe the persistence of small cell foci in about 25% of the animals that persisted temporarily for more than 7 days [24], suggesting that these cells have the capacity to lodge and proliferate in non-target tissue sites. If they do proliferate, this might create a long-term hazard. There will therefore be a need either to identify an MSC source that is effective therapeutically but that does not carry this risk, or to engineer the cells to have a genetic deficiency such that they can thrive only in the presence of a nutrient or other small, harmless molecule not normally found in humans. When regeneration is over, this could be withdrawn so that the introduced MSC cells die. Much better still would be to replace the need to introduce cells through the development of pharmacological mimics of their presence.

## The goal of producing replacement tissues

An alternative to promoting the regeneration of organs and tissues *in situ* is to grow a new organ *in vitro* from the patient’s stem cells (perhaps with any genetic defect corrected), and then to transplant it back to the patient either as a full-sized organ or an embryonic-sized one that will grow *in situ*. For the kidney, progress towards this began in 2010 with a demonstration that a suspension of cells from a disaggregated mouse embryonic kidney rudiment (which includes stem cells) would, when re-aggregated, go on to develop nephrons and collecting duct tubules [25]. This showed that the cells had the power to self-organize. It was striking, however, that while the micro-anatomy of the resulting organoids was realistic compared to a late foetal kidney, the macro-anatomy of the organ was entirely absent. This type of organization, with realistic tubules arranged haphazardly, has become known informally in the field as a ‘bag-of-socks’. In 2015, two groups developed methods for differentiating human induced pluripotent stem (hiPS) cells towards renal fates and produced human renal organoids with a similar micro-anatomy-correct, macro-anatomy-missing, bag-of-socks level of organization [15,26]. These were originally produced manually and in small numbers, but subsequent work has developed high-throughput methods (e.g. [27]).

Production of hiPS-derived organoids is plagued with serious inter-experiment variability, and a recent analysis has now shown that most of this variability arises between, rather than within differentiation runs, and is associated with different proportions of cell types from each run [28]. These differences in outcome between differentiation runs are greater than those between different hiPS lines, and include generation of non-renal cell types as well as the renal ones intended [29]. One approach to addressing variability has been to move away from the idea of differentiating all necessary stem cells together and letting the mix develop, towards methods in which different stem cell types are generated separately and combined in defined proportions [30^•^,^31^]. Protocols for generating specific stem cell types (e.g. nephron progenitor cells, NPC, and Ureteric Bud Progenitor Cells, UBPC) have been developed (e.g. [32, 33, 34]). Some of these cell types, once generated, can also be expanded in culture [35,36].

**Figure 2:**
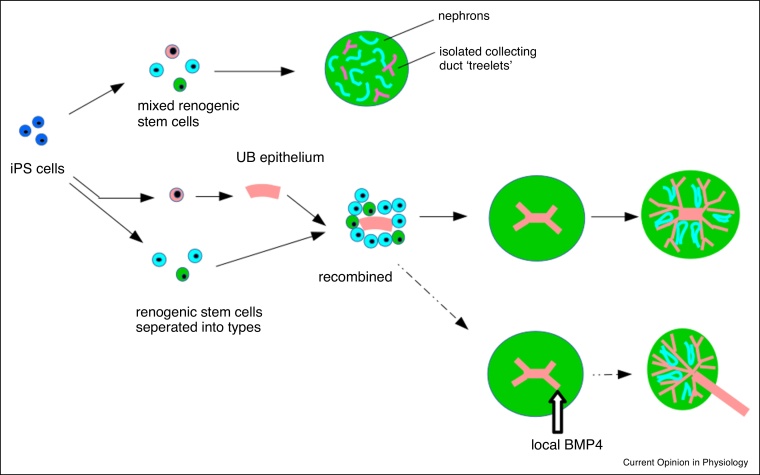
Self-organization in renal organoids, and the importance of symmetry-breaking for large-scale anatomical realism. The top row shows the first-developed method for making renal organoids, in which iPS cells are differentiated into mixed renogenic stem cells (e.g. nephron precursors shown in blue, stromal precursors in green and ureteric bud precursors in pink). The cells organize themselves into individually realistic tubules but there is no realistic gross anatomy. The middle row begins with generation of separated stem cells groups (different protocols do this to different extents); generation of a ureteric bud (UB) epithelium, and then mixing it with the other cell types, breaks the symmetry of the system by including one unique, local source of ureteric bud. This develops into a single, connected tree, and nephrons organize around it appropriately. The bottom row shows a possible way of improving realism still by breaking the symmetry of the ureteric tree using local BMP application; this works in organoids made from ex-fetu renogenic stem cells but has not yet been done with iPS-derived ones.

Organoids made by these protocols will, if transplanted into mammalian hosts, become vascularized and show basic filtration of blood, and this helps them mature more than they do *in vivo* alone [16,37,38]. But they cannot work as replacement organs, partly because of their size but mostly because they do not feature correct large-scale organization (connection of nephrons to a single collecting duct tree, connected in turn to a ureter, and a corticomedullary axis), which is crucial for normal renal function. Work in organoids made from renogenic stem cells, obtained from embryonic mouse kidney rudiments, has indicated that large-scale organization can be imposed on organoids by externally applied symmetry-breaking (Figure 2). Introducing ureteric bud stem cells as an intact epithelium, rather than as scattered individuals, into the mix of other stem cells results in the organization of the organoid around a single collecting duct tree, and correct nephron orientation and cortico-medullary organization [39,40]. This idea has been transferred to ES cell-derived organoids, and works [30^•^]. In organoids made from ex-fetu cells, a further imposed break of symmetry from a local source of BMP7 can convert one nearby developing collecting duct into a urothelial exit tube, leaving the other tubes to make the collecting duct and the nephrons to arrange around that [41^•^].

It must be stressed that, even if the ureter-making technique transfers to hiPS-derived organoids to give them large-scale organization, they will still be a long way from usable kidneys. A blood system will have to be introduced (the anatomy of the blood system will also have to be realistic; the relation of its vessels to the corticomedually organization of the kidney is critical to function). Also, the organ will have to be three-dimensional rather than flat (as most organoids are), and it needs to be much larger. The latter may be easier to achieve by grafting a small organ into the final host: human kidney rudiments have successfully been transplanted into rats, where they grow in size and can support the life of nephrectomized rats for months [42^•^]. If iPS-derived organoids could be made to the stage of maturation of these human organ rudiments, they might also be able to grow in hosts.

## Marketing, hope and hype

Considerable progress has been made to lay foundations for renal regenerative medicine, but this work has not yet been translated into a clinical discipline. We are beginning to understand the mechanisms whereby exogenous cells promote the repair of renal tissue, and the advances made towards generating functional renal tissue from stem cells have been extraordinary. However, as highlighted above, much more work is needed before any of these approaches could be used in patients. Despite this, overly positive media articles often give the impression that stem cell therapies for various diseases are just around the corner. An unfortunate consequence of this is that many patients now have unrealistic expectations of what can currently be achieved, and this has helped fuel a ‘for-profit’ direct-to-consumer (DTC) market for unproven ‘stem’ cell therapies. The problems associated with DTC marketing have been known for many years, with the risks and financial burden posed to patients being highlighted in a report from Caulfield’s group in 2008 [43]. Since then, however, there has been a burgeoning growth in this industry, particularly in the US where the number of clinics increased from 2 to almost 600 between 2008 and 2016 [44]. A detailed investigation by Rasko *et al.* has shown that while DTC stem cell clinics were initially restricted to developing economies with poor regulatory oversight, they are now prevalent in the US, Australia, and several EU countries including the UK, Germany and Ireland [45].

The increase in market demand for unproven stem cell therapies, despite warnings from academics and professional bodies, is probably due to ‘scienceploitation’, a term coined by Caulfield to describe the phenomenon of creating false and/or misleading information about a legitimate area of science [46^•^]. Journalists may engage in stem cell scienceploitation unintentionally due to lack of expert knowledge, and/or insufficient time to check the validity of stories about potential stem cell cures, the source of which can often be University press offices [47]. In contrast, stem cell banks and clinics may use scienceploitation intentionally to boost consumer interest in their products and services [46^•^]. If unchecked, the scienceploitation of stem cell research risks harming patients, damaging public trust and bringing the field into disrepute.

Apart from the problems with misleading information, it is also important to consider the ethical and legal issues associated with the marketing of unproven stem cell therapies. Some practices would probably be regarded as unethical, but may still be legal. For instance, while it is currently illegal for clinics in the UK to sell unproven therapies for serious conditions such as Parkinson’s and heart disease, they can still facilitate these practices by providing cells to clinics based in countries where there is less regulatory oversight. Such an arrangement existed between the UK stem cell banking company, ‘Precious Cells’ (which entered into administration in 2018), and the Lebanon-based stem cell clinic, ‘Cells4Life’ [48]. It may also be the case that some banks/clinics are contravening trading standards legislation [46^•^]. For example, a Human Tissue Authority inspection report of a UK company that banks MSCs revealed that the preparation process dossier submitted by the company’s outsource partner did not demonstrate the presence of MSCs [49]. It can thus be seen that this company did not appear to be complying with UK trading standards but was nevertheless able to operate.

To address these issues, it would be helpful to define the key ethical concerns relating to the DTC marketing of stem cell therapies, identify the laws that could potentially impact their regulation and work with policy-makers towards improving legislation to curtail unethical practices. Without such action, there is a real danger that any potential future benefit of stem cell and regenerative medicine research will be overshadowed by the harmful practices of unscrupulous companies.

## Conflict of interest statement

Nothing declared.

## References and recommended reading

Papers of particular interest, published within the period of review, have been highlighted as:

• of special interest
